# Lifestyle factors and prevalence of semen abnormalities among men undergoing infertility evaluation at oak specialist hospital: A retrospective cohort study

**DOI:** 10.1371/journal.pone.0340902

**Published:** 2026-01-15

**Authors:** Rex Mawuli Kwadjo Djokoto, Victor Boachie Owusu, Edward Anabila Agana, Kingsley Afreh Nduroh, Johnny Arthur-Komeh, Opei Adarkwa, Andrew Panyin Vormawor, Anthony Amanfo Ofori, Isaac Kofi Adu

**Affiliations:** 1 Kwame Nkrumah University of Science and Technology, Obstetrics and Gynaecology, Kumasi, Ghana; 2 Oak Specialist Hospital, Obstetrics and Gynaecology, Kumasi, Ghana; 3 Kwame Nkrumah University of Science and Technology, Physiology, Kumasi, Ghana; 4 Komfo Anokye Teaching Hospital, Obstetrics and Gynaecology, Kumasi, Ghana; 5 Powerhouse Hospital, Obstetrics and Gynaecology, Kumasi, Ghana; 6 Manhyia Government Hospital, Obstetrics and Gynaecology, Kumasi, Ghana; Beijing Normal University, CHINA

## Abstract

**Background:**

Male infertility constitutes a notable health burden in Sub-Saharan Africa, contributing to approximately 40–50% of all infertility cases, compounded by cultural stigma and a grave knowledge issue. The study aimed to apply the updated World Health Organisation (WHO) 6th Edition (2021) criteria to a Ghanaian cohort, generating contemporary prevalence data on semen abnormalities.

**Methods:**

A retrospective cohort study design was employed, analysing the medical and laboratory records of 221 men who underwent semen analysis as part of an infertility investigation at Oak Specialist Hospital, Kumasi, Ghana. A consecutive sampling method was used to include all eligible patient records from September 1, 2022, to August 31, 2024. Key variables included semen parameters (volume, concentration, motility, morphology, leukocytes) analysed per WHO 2021 guidelines, and associated lifestyle and demographic factors. Data were analysed using descriptive statistics, prevalence rates, and regression analysis.

**Results:**

The study identified high prevalence rates of oligozoospermia (38.91%) and teratozoospermia (38.46%). A critically high prevalence of leukocytospermia was the most striking finding, affecting 80.54% of the cohort. Regression analysis revealed significant negative associations between sugary drink consumption and sperm motility (p = 0.038) and a positive association between caffeine intake and sperm morphology (p = 0.011). Paradoxically, the cohort’s median values for semen volume, concentration, and motility were significantly above the WHO 2021 lower reference limits.

**Conclusion:**

Using current international standards, this study provides updated baseline data on male infertility patterns in urban Ghana. The findings reveal a high burden of specific abnormalities, particularly in sperm morphology and leukocyte concentration, which may be more clinically relevant than simple concentration or motility deficits in this population. Modifiable lifestyle factors, such as diet, represent viable targets for public health intervention. These results are vital for clinical benchmarking, guiding targeted health strategies, and establishing a foundation for future comparative studies in similar resource-constrained settings.

## Introduction

Infertility is a significant global health issue, affecting an estimated 15% of couples of reproductive age worldwide [1]. The contribution of male factor infertility to this burden is substantial, accounting for approximately 40–50% of all cases and representing a major, often overlooked, component of reproductive health [2]. While this challenge is universal, its prevalence, societal impact, and research focus vary considerably across regions. In Sub-Saharan Africa, infertility transcends a mere individual clinical concern to become a profound public health problem, frequently exacerbated by under-reporting, limited access to care, and a lack of robust research infrastructure [3]. Compared to the extensive body of work on female fertility in the region, male fertility remains a relatively understudied domain, creating a significant void in public health knowledge and clinical practice [4].

This knowledge breach is particularly pertinent in Ghana, where infertility is a culturally sensitive and often stigmatising issue. Societal pressures and traditional norms frequently result in the burden of blame being disproportionately placed on women, which can lead to marital instability and psychological distress [5]. However, emerging evidence from Ghana points towards a concerning and escalating trend of declining male reproductive health. A landmark retrospective study conducted in Tema from 1995 to 2005 revealed a stark decline in semen quality over the decade. The proportion of men with normal sperm concentration fell precipitously, while the prevalence of oligozoospermia (low sperm count) more than doubled, increasing from 20.5% to 57.6% [6]. Similarly, asthenozoospermia (poor sperm motility) became markedly more common. A study by Gyasi-Sarpong et al. (2017) from Kumasi, Ghana’s second-largest city, corroborate this trend where they identified high rates of oligozoospermia (44.20%), azoospermia (31.97%), and other sperm parameter abnormalities among men attending a fertility clinic [3]. These findings collectively underscore a potential escalating crisis in male reproductive health in urban Ghanaian settings that demands urgent and contemporary investigation.

The clinical cornerstone for diagnosing male infertility is semen analysis, a laboratory test that evaluates key sperm characteristics, including concentration (count), motility (movement), and morphology (shape), against established reference values [6]. For decades, the World Health Organisation (WHO) has published comprehensive manuals to standardise this process globally, ensuring data comparability across different laboratories and regions. A pivotal development in this field is the recent publication of the WHO Laboratory Manual for the Examination and Processing of Human Semen, 6th Edition [7]. This updated manual expands on the 5th Edition by combining its data with a new cohort of 1,789 fertile men, thereby addressing previous demographic under-representation and providing updated 5th percentile lower reference limits (LRLs) for key parameters. For instance, the 5th percentile LRL for semen volume is 1.4 mL, total motility is 42%, and progressive motility is 30% [8]. The 6th Edition emphasizes that these LRLs are not rigid diagnostic thresholds but rather decision guides for clinical interpretation, encouraging a holistic evaluation of patient pathology rather than reliance on isolated values [9]. This update assists in standardizing diagnosis, monitoring treatment, and guiding clinical choices in assisted reproduction.

The aetiology of declining semen quality is multifactorial, with a growing body of evidence implicating modifiable lifestyle and environmental factors. Research conducted specifically in Ghana has established significant links between such factors and poor semen quality. Studies have shown that smoking, psychological stress, elevated body mass index (BMI), and the consumption of certain alcoholic beverages are associated with reduced sperm concentration, motility, and viability [10]. This provides a regional basis for this study’s objective to explore correlations between patient characteristics and semen parameters, moving beyond simple prevalence to investigate potential drivers of infertility.

The justification for this study is born from the confluence of these critical factors: (1) the historical evidence of deteriorating semen quality in major Ghanaian cities, suggesting a public health problem of increasing magnitude [10]; (2) the recent publication of updated WHO (2021) diagnostic criteria have not yet been widely applied in this context, making current data essential for international comparison; and (3) a lack of current data from a major specialist fertility centre in Kumasi that utilizes these new standards. The study is therefore designed to fill this critical knowledge gap. It seeks to be one of the first application of the WHO 2021 criteria to a cohort of men undergoing fertility evaluation in urban Ghana. In doing so, the study generates a precise, contemporary, and clinically relevant baseline of abnormality prevalence and its associated factors. This will not only provide a truer picture of the current public health burden within this clinical setting but also establish a vital benchmark for future clinical practice, public health interventions, and comparative research across West Africa.

## Methodology

### Research approach

#### Study design.

A retrospective cohort study design was employed. This observational design involved identifying a cohort of men undergoing fertility evaluation who attended Oak Specialist Hospital, and analysing their pre-existing medical and laboratory records to examine exposures (e.g., age, lifestyle factors) and outcomes (semen parameters) that occurred in the past [11]. This design was chosen for its primary advantages of time and cost efficiency, as its utilised data that had already been systematically collected. It allowed for the analysis of a large volume of data on numerous subjects, which was advantageous for establishing accurate prevalence rates and exploring statistical correlations with sufficient power [12]. To mitigate the inherent limitations of retrospective studies, such as the potential for missing data, a clear protocol was established to exclude records with incomplete data on primary outcome variables.

#### Study area.

The study was conducted at Oak Specialist Hospital, a private healthcare facility located in Fankyenebra, a suburb within the Kumasi Metropolis of the Ashanti Region, Ghana [13]. As Ghana’s second-largest city, Kumasi has a large and diverse urban population, making it a representative setting for studying urban health trends in West Africa [14]. The hospital is a leading centre for assisted reproductive techniques (ART), which provides a concentrated and accessible population of the target subjects. Data were collected from records created during the period spanning September 1, 2022, to August 31, 2024.

#### Study population.

The target population for the study comprises all complete sets of medical records for all male patients, aged between 20 and 50 years, who underwent a semen analysis as part of an infertility investigation at Oak Specialist Hospital between September 1, 2022, and August 31, 2024 and that data extraction from the hospital’s EMR occurred between October 1, 2024 and February 20, 2025. This defined two-year period is expected to yield a sufficiently large dataset for strong statistical analysis.

While the population is drawn from a private specialist facility, it is highly representative of the specific, clinically important subgroup of couples who actively seek medical intervention for infertility in the region. The findings will thus be directly generalizable to this clinical population, providing valuable insights into the characteristics of men engaging with the healthcare system for fertility issues.

### Sampling method

A consecutive sampling technique was utilised, in which every patient record from the accessible population that met the predefined inclusion criteria was included in the research sample [15]. This census approach was adopted to maximise the sample size, minimise selection bias, and ensure the sample was as representative as possible of the clinical population served by the hospital during that timeframe. This method resulted in a final cohort of N = 221.

#### Selection criteria.

The inclusion criteria for the study consisted of medical records of male patients aged 20–50 years who underwent a complete semen analysis at Oak Specialist Hospital, with a clinical notation or diagnosis in their records indicating an investigation for fertility evaluation.

#### Exclusion criteria.

The exclusion criteria included records with incomplete or missing data for the primary semen parameters, namely sperm concentration, motility, and morphology. Additionally, patients with a documented history of vasectomy or other known obstructive causes of azoospermia, as well as those with known genetic disorders established as causes of severe spermatogenic failure, such as Klinefelter syndrome, were excluded from the study.

### Definitions of semen parameters and abnormalities

All semen parameters were classified according to the WHO 6th Edition (2021) lower reference limits (LRLs) (7). Terminology for abnormalities was defined as follows:

#### Semen volume.

Classified as low (Hypospermia) if < 1.4 mL.

#### Sperm concentration.

Classified as low (Oligozoospermia) if < 16 million/mL.

#### Total motility.

Classified as low (Asthenozoospermia) if < 42%.

#### Progressive motility.

Sperm Morphology Classified as low (Teratozoospermia) if < 4% normal forms.

#### Leukocytospermia.

Defined as the presence of leukocytes (pus cells) at a concentration > 1.0 million/mL.

#### Combined abnormalities.

Defined as Oligoasthenozoospermia (low concentration and motility) and Oligoasthenoteratozoospermia (OAT; low concentration, motility, and morphology).

### Data collection method

Data were collected retrospectively between October 1, 2024 and February 20, 2025 by accessing the hospital’s secure Electronic Medical Records (EMR) system and the associated Laboratory Information System (LIS). A standardised data extraction form, developed in Microsoft Excel, was used to ensure that data collection was systematic and consistent across all records. This instrument enhanced the reliability and validity of the final dataset [12]. Variables extracted included demographics, lifestyle factors, and macroscopic and microscopic semen parameters based on the WHO 6th Edition (2021) guidelines. All data were de-identified at the point of extraction by assigning a unique, non-identifiable study ID to maintain patient confidentiality.

### Data analysis

All statistical analyses were conducted using STATA software, version 17. The extracted data were cleaned and checked for errors in Microsoft Excel before importation into STATA. The distribution of continuous variables was assessed for normality using the Shapiro-Wilk test.

Given that the WHO 2021 semen parameter thresholds represent the lower 5th percentile values of fertile men, and are intended solely to guide individual-level clinical decision-making, they were not treated as population reference means or medians for inferential comparison. For each semen parameter, we calculated the proportion of participants falling below the WHO 2021 lower reference limit, together with exact 95% confidence intervals, to describe the burden of sub-threshold values within the cohort.

To examine determinants of semen quality, multivariable regression analysis was conducted with semen parameters as dependent variables and patient characteristics (e.g., age, BMI, abstinence duration, lifestyle factors, and medical history) as independent variables. Because semen parameters are continuous, non-binary outcomes, we applied multivariable linear regression models. Results are reported as regression coefficients (β) with their corresponding 95% confidence intervals (CI). In this context, a β-coefficient CI not including 0 was interpreted as statistically significant, consistent with the null hypothesis value of 0 for linear regression coefficients.

A significance level of p < 0.05 was applied for all inferential tests. All analytical decisions were guided by the clinical purpose of the WHO 2021 thresholds and the methodological requirement for appropriate regression interpretation.

### Ethics application

Ethical approval was obtained from the Committee on Human Research, Publication and Ethics (CHRPE), School of Medical Sciences, Kwame Nkrumah University of Science and Technology (KNUST)/Komfo Anokye Teaching Hospital, with the reference number CHRPE/AP/1050/24. Formal written permission was also secured from the management of Oak Specialist Hospital. A waiver of individual informed consent was granted by the CHRPE, which is standard practice for minimal-risk retrospective chart review research. The principle of patient confidentiality was paramount; all data were de-identified, analysed in an aggregated format, and stored securely in password-protected files to prevent the identification of any individual, in adherence with the Data Protection Act (Act 843), 2012 of Ghana.

## Results

### Socio-demographic and anthropometric characteristics of participants

Table 1 below presents the socio-demographic and anthropometric of the study participants. The median (interquartile range) age was 40 years (34–46), with slightly more participants aged 40 years and below (53.39%) compared to those above 40 years (46.61%). The median waist circumference was 36 inches (34–39), with over half (52.04%) measuring 36 inches or less. Hip circumference had a median of 40 inches (32–43), with 54.75% recording 40 inches or below. The majority of participants were married (97.74%), non-smokers (96.83%).

**Table 1:** a. Socio-demographic and anthropometric characteristics of participants.

Variables	Frequency (N)	Percentage (%)
Age (years)	M(IQR): 40(34,46)	
40 and below	118	53.39
Above 40	103	46.61
Waist Circumference	M(IQR): 36(34,39)	
36 and below	115	52.04
Above 36	106	47.96
Hip Circumference	M(IQR): 40(32,43)	
40 and below	121	54.75
Above 40	100	45.25
Marital Status		
Single	5	2.26
Married	216	97.74

**Source: Field data.**

**Data are expressed as percentages and frequencies. M: Median scores, IQR: 25**^**th**^ **and 75**^**th**^ **Interquartile.**

### Lifestyle and environmental characteristics of participants

Table 2 presents the lifestyle and environmental characteristics of the 221 participants. The majority of participants reported non-smoking status (96.83%), and almost half had never consumed alcohol (47.51%), although a substantial proportion consumed alcohol sometimes (42.08%). Caffeine intake followed a similar pattern, with nearly half of participants never consuming caffeinated drinks (48.87%), while 37.10% consumed them sometimes. Regarding physical activity, most participants engaged in light (33.03%) or sedentary (31.67%) activity levels, with fewer reporting moderate (25.79%) or vigorous (9.50%) exercise. Consumption of sugary foods and drinks was common, with nearly half (49.77%) reporting intake sometimes, and 25.79% consuming them often. Environmental and device-related exposures varied: over half (57.92%) frequently carried their phones in the groin area, while 69.23% never used laptops on their laps. Sitting time was relatively high, with 42.99% sitting for 1–4 hours daily and 42.53% for more than 4 hours. In terms of occupational exposures, 22.62% reported exposure to radiation, while 46.61% had some level of industrial chemical exposure. Engagement in outdoor pollution-related activities was also notable, with 48.87% participating sometimes, often, or always, although over half (51.13%) reported no involvement in such activities.

**Table 2:** b. Lifestyle and environmental characteristics of participants.

Variables	Frequency (n)	Percentage (%)
Smoking status		
No	214	96.83
Yes	7	3.17
Alcohol consumption		
Never	105	47.51
Sometimes	93	42.08
Often	15	6.79
Always	8	3.62
Caffeina**ted** drink consumption		
Never	108	48.87
Sometimes	82	37.10
Often	22	9.96
Always	9	4.07
Routine exercise level		
Sedentary (little/no activity)	70	31.67
Light (occasional walking/light activity)	73	33.03
Moderate (regular exercise, e.g., jogging)	57	25.80
Vigorous (intense workouts, weightlifting)	21	9.50
Sugary food and drink consumption		
Never	28	12.67
Sometimes	110	49.77
Often	57	25.79
Always	26	11.77
Phone in pocket near groin area		
No	39	17.65
Sometimes	54	24.43
Yes	128	57.92
Use of laptops on laps		
Never	153	69.23
Rarely	48	21.72
Occasionally	14	6.34
Regularly	6	2.71
Daily sitting hours		
< 1 hour	32	14.48
1–4 hours	95	42.99
> 4 hours	94	42.53
Work exposure to radiation		
No	171	77.38
Yes	50	22.62
Exposure to industrial chemicals		
Never	118	53.39
Sometimes	67	30.32
Often	27	12.22
Always	9	4.07
Engagement in outdoor pollution activities		
Never	113	51.13
Sometimes	72	32.58
Often	19	8.60
Always	17	7.69

**Source: Field data.**

### Distribution of semen parameters (volume, PH, concentration, total count, total motility, progressive motility, and normal morphology)

Table 3 presents the distribution of semen parameters among the study participants. The median (IQR) semen volume was 2.0 ml (1.5–3.0), with most participants (75.57%) having normal volume, and 24.43% showing low values. Sperm concentration had a median of 23 million/ml (0.1–71), with 61.09% in the normal range, and 38.91% low. Median total motility was 50% (35–60), with the majority (73.30%) having normal motility and 26.70% abnormal. Progressive motility had a median of 50% (40–65), with all participants (100%) falling into the high category. Sperm morphology had a median of 5% (2–11), with 61.54% normal and 38.46% abnormal. Pus cell leukocyte concentration had a median of 2 million/ml (2–4), with a notable 80.54% of participants showing abnormal levels and only 19.46% within the normal range. These indicate that while most semen parameters were within normal limits for the majority, high rates of abnormal leukocyte concentration and reduced sperm morphology were observed. **(Table 3)**

**Table 3:** Distribution of semen parameters and classification based on WHO 2021 lower reference limits (LRLs).

Variables	WHO 2021 Threshold (LRL)	Frequency (N)	Percentage (%)
Semen volume (ml)	<1.4 mL (Low)	M(IQR): 2.0(1.5,3)	
Low		54	24.43
Normal		167	75.57
Sperm count (mill/ml)	<16 million/mL (Low)	M(IQR): 23(0.1,71)	
Low		86	38.91
Normal		135	61.09
Sperm Motility (%)	<42% (Low)	M(IQR): 50(35,60)	
Low		71	32.13
Normal		150	67.87
Progressive motility (%)	<30% (Low)	M(IQR): 50(40,65)	
Low		0	0.00
Normal		221	100.00
Sperm Morphology (%)	<4% (Abnormal)	M(IQR): 5(2,11)	
Abnormal		85	38.46
Normal		136	61.54
Pus cell Leukocytes (mil/ml)	≥1 million/mL (Abnormal)	M(IQR): 2(2,4)	
Abnormal		178	80.54
Normal		43	19.46

**Source: Field data.**

*WHO 2021 lower reference limits (LRLs) represent the lower 5th percentile of fertile men with time-to-pregnancy < 12 months. Values below these limits indicate potential clinical concern. Asthenozoospermia is defined as total motility <42% or progressive motility <30% in accordance with WHO 6th Edition.*

### Prevalence of combined semen abnormalities based on WHO 6th edition (2021) lower reference limits

Table 4 presents the prevalence of combined semen abnormalities in the study cohort based on the WHO 6th Edition (2021) lower reference limits. The findings show that oligoasthenozoospermia, defined as the co-occurrence of low sperm concentration and reduced motility, was identified in 48 participants, representing 21.72% of the cohort. A smaller proportion of participants exhibited oligoasthenoteratozoospermia (OAT) the simultaneous presence of low sperm concentration, reduced motility, and abnormal morphology with 6 cases, accounting for 2.71% of the sample. These results indicate that while single-parameter abnormalities were common, a substantial subset of men experienced multiple concurrent impairments in semen quality, highlighting the clinical relevance of assessing combined semen abnormalities when evaluating male fertility potential

**Table 4:** Prevalence of combined semen abnormalities based on WHO 6th edition (2021) lower reference limits.

Abnormality	Frequency (N)	Percentage (%)
Oligoasthenozoospermia	48	21.72
Oligoasthenoteratozoospermia (OAT)	6	2.71

Source: Field data.

### Association between key semen parameters (concentration, motility, morphology) and patient characteristics available in the records

Table 5 summarises the multivariable regression analysis examining associations between patient characteristics and semen quality indicators. Most predictors showed no statistically significant associations with sperm motility, count, or morphology. However, a few notable relationships were observed. In addition, age was associated with a β = −0.04 mL/year reduction in semen volume (95% CI −0.06 to −0.01; p = 0.001), indicating a small but statistically significant decline per year. Higher reported caffeine intake was associated with higher normal morphology (β = 0.68; 95% CI 0.16 to 1.20; p = 0.011). Given the observational design and potential residual confounding, this finding is exploratory and should not be interpreted causally. Intake of sugary food was associated with lower total motility (β = −2.27; 95% CI −4.42 to −0.12; p = 0.038). Regular laptop-on-lap use was positively associated with sperm concentration (β = 7.05 million/mL; 95% CI 1.74 to 12.36; p = 0.010). Industrial exposure and outdoor activity did not demonstrate any statistically significant relationships with semen quality. The models indicates that while most lifestyle and environmental exposures were not significant predictors, specific factors such as caffeine intake, sugary food and laptop placement were significantly associated with some semen parameters such as morphology, motility, and count respectively.

**Table 5:** Association between key semen parameters (concentration, motility, morphology) and patient characteristics available in the records, specifically age.

	Sperm Motility		Sperm Count		Sperm Morphology	
Predictor	Coef.(95%CI)	p-value	Coef.(95%CI)	p-value	Coef.(95%CI)	p-value
Age	−0.08(−0.42, 0.26)	0.625	−0.04(0.66, 0.59)	0.913	−0.01(−0.09, 0.07)	0.845
Waist circumference	0.28(−0.43, 0.99)	0.433	−0.28(−1.59, 1.02)	0.670	−0.06(−0.23,0.11)	0.497
Hip circumference	−0.04(−0.36, 0.28)	0.817	0.40(−0.18, 0.99)	0.178	−0.01(−0.09, 0.06)	0.704
Smoking (yes)	0.80(−13.95, 15.54)	0.915	8.27(−18.83, 35.37)	0.548	1.05(−2.49, 4.60)	0.558
Alcohol (yes)	0.52(−1.25, 2.29)	0.562	0.49(−2.76, 3.75)	0.766	−0.25(−0.68, 0.17)	0.239
Caffeine intake	−1.58(−3.75, 0.60)	0.154	−2.04(−6.04, 1.95)	0.314	0.68(0.16, 1.20)	**0.011**
Exercise routine	−1.15(−3.75, 1.44)	0.382	−4.60(−9.37, 0.17)	0.059	0.02(−0.60, 0.65)	0.945
Sugary food	−2.27(−4.42, −0.12)	**0.038**	0.87(−3.08, 4.81)	0.666	0.12(−0.39, 0.64)	0.637
Phone in pocket	−1.62(−5.04, 1.80)	0.351	0.09(−6.19, 6.37)	0.978	−0.02(−0.84, 0.80)	0.969
Laptop on lap	−0.21(−3.10, 2.68)	0.885	7.05(1.74, 12.36)	**0.010**	0.35(−0.34, 1.04)	0.321
Sitting daily	1.90 (−0.84, 4.64)	0.173	−1.01(−6.05, 4.03)	0.692	−0.37(−1.02, 0.29)	0.275
Work exposure	3.14 (−3.17, 9.46)	0.327	10.52(−1.08, 22.12)	0.075	−0.36(−1.87, 1.16)	0.642
Industrial exposure	0.21 (−1.96, 2.38)	0.847	1.38(−2.61, 5.36)	0.497	−0.31(−0.83, 0.21)	0.246
Outdoor activity	−0.87 (−2.91, 1.17)	0.401	−0.16(−3.92, 3.59)	0.931	0.07(−0.42, 0.56)	0.772

### Cohort distribution relative to WHO 2021 Lower Reference Limits (LRLs)

Table 6 compares the cohort’s semen parameters to the WHO 2021 lower reference limits (LRLs). Although the median values for volume, concentration, motility, and morphology were above the WHO thresholds, a considerable proportion of participants still fell below these clinical cut-offs. Specifically, 42.86% had low semen volume, and 43.75% had sperm concentration below 16 million/mL. One in five men (20.00%) had total motility below the 42% threshold, while 25.00% showed morphology below 4%. Leukocytospermia was present in all participants, with 100% exceeding the WHO limit of >1 million leukocytes/mL. These findings highlight the distribution of semen characteristics in the cohort and identify the proportion of men who may require further clinical evaluation.

**Table 6:** Cohort distribution relative to WHO 2021 Lower Reference Limits (LRLs).

Sperm Parameter	WHO 2021 Reference	Cohort Median (IQR)	% Below WHO LRL
Volume (mL)	1.4	2.0 (1.5,3)	42.86
Concentration (mill sperm/mL)	16 million/mL	23 (0.1,71)	43.75
Motility (%)	42%	50 (35,60)	20.00
Normal Morphology (%)	4%	5 (2,11)	25.00
Leukocyte (mill/mL)	<1 million/mL	2 (2,4)	100

**Source: Field data.**

## Discussion

### Prevalence of semen abnormalities

The study applied the WHO 6th Edition (2021) criteria to a cohort of men undergoing fertility evaluation in Ghana demonstrates heterogeneity in male reproductive health indicators. The principal finding is a striking dichotomy: while median values for several parameters (e.g., concentration, motility) were above the LRLs, the prevalence of specific, severe abnormalities was high. Specifically, the rates of isolated teratozoospermia (38.46%) and, most critically, leukocytospermia (80.54%) were profound. This suggests that for a large proportion of infertile men in this urban setting, the primary issue may not be a simple deficit in sperm quantity or movement, but rather a more fundamental problem with sperm quality (morphology) and the health of the seminal milieu (inflammation).

When contextualised against historical local data, these findings provide new insights. The oligozoospermia prevalence of 38.91% in our study is lower than the 57.6% reported in Tema (1995–2005) [5] and the 44.20% in Kumasi before 2017 [3]. A direct comparison of prevalence rates across different diagnostic eras is challenging, as these earlier studies used different WHO criteria (e.g., 5th Edition LRL of 15 million/mL or older 20 million/mL limits). The application of the 6th Edition’s 16 million/mL LRL may reclassify some men, making direct prevalence trend analysis difficult. Conversely, the high rate of teratozoospermia (38.46%) identified using the 4% normal morphology cutoff of the 6th Edition likely represents a more accurate detection of morphological defects compared to older, less rigorous assessment methods. The data challenge the simplistic narrative of a universal decline in sperm count, as the median concentration of 23 million/mL is well above the WHO LRL. However, the high prevalence of oligozoospermia indicates a highly skewed distribution with a substantial subgroup of men having very low counts. The real story emerging from this cohort is not about the average man’s sperm count, but about the significant proportion of men with severe deficits in sperm quality and seminal health, pointing toward aetiologies that go beyond spermatogenic efficiency alone.

The more salient deduction is that the application of modern criteria reveals a specific pattern of dysfunction. The data challenge the simplistic narrative of a universal decline in sperm count. The median concentration of 23 million/mL is well above the WHO cutoff, but the high prevalence of oligozoospermia indicates a highly skewed distribution with a substantial subgroup of men having very low counts. The real story emerging from this cohort is not about the average man’s sperm count, but about the significant proportion of men with severe deficits in sperm quality and seminal health, pointing toward aetiologies that go beyond spermatogenic efficiency alone.

### The impact of lifestyle factors on semen quality

The study identified several statistically significant associations, though these must be interpreted with extreme caution given the study’s retrospective and cross-sectional nature. The negative association between higher consumption of sugary foods and drinks and sperm motility (Coef. = −2.27, p = 0.038) is a relevant finding. This aligns with the well-documented nutrition transition occurring in urban centres across Ghana, where traditional diets are increasingly replaced by processed foods and sugar-sweetened beverages [16]. The biological plausibility for this association is strong; which suggest that sugar intake may play a contributory role in impaired sperm motility through systemic metabolic pathways.

More unexpectedly, a positive association was found between higher caffeine intake and better sperm morphology (Coef. = 0.68, p = 0.011) and between regular laptop use on the lap and higher sperm count (Coef. = 7.05, p = 0.010). These findings are counter-intuitive and contradict some, though not all, of the existing literature [17]. It is critical to note that these are merely statistical associations from self-reported data. They may be the result of unmeasured confounding variables (e.g., socioeconomic status, recall bias, or other lifestyle factors not captured in the records).

### The leukocytospermia enigma

The most conspicuous and clinically pressing discovery of this our study is the extraordinarily high prevalence of leukocytospermia (80.54%), defined as a leukocyte concentration of >1.0 × 10^6^ white blood cells per mL of semen. This rate is dramatically higher than the 5–20% prevalence typically reported in infertile men globally [18] and may represent the single most important, under-reported, and potentially treatable cause of male infertility in this population in Ghana. Indeed, the cohort’s median leukocyte count was 2.0 million/mL (IQR 2–4), double the WHO threshold, indicating this is a widespread and significant inflammatory issue within this population, not an outlier. Leukocytospermia is a direct marker of inflammation or infection within the male genitourinary tract. The presence of activated leukocytes in semen leads to the excessive production of reactive oxygen species (ROS), which are known to inflict severe damage on sperm membranes, DNA integrity, and motility. Therefore, this single finding could be the underlying pathophysiological driver for a large proportion of the other semen abnormalities observed in this cohort, such as poor motility and morphology.

This outcome suggests a potential paradigm shift in the diagnostic and therapeutic approach to male infertility in this setting, away from a focus on idiopathic spermatogenic failure or expensive assisted reproductive technologies, and towards the investigation and treatment of underlying inflammation and infection. The potential local causes for this high rate are speculative but demand investigation. They may include a high community prevalence of undiagnosed and asymptomatic genitourinary infections such as prostatitis or urethritis, including sexually transmitted infections [9]. Environmental factors, such as exposure to specific local pollutants that induce an inflammatory response, or cultural practices related to genital hygiene or clothing that might predispose men to infections, could also play a role [10]. This finding represents a substantial and previously under-reported potential cause of infertility in this cohort that warrants urgent clinical attention and dedicated potential investigation to confirm its prevalence and identify its underlying aetiology. A simple, low-cost intervention, such as a course of antibiotics or anti-inflammatory agents for a confirmed underlying condition, could potentially resolve infertility for a significant number of these men, making this a high-yield area for clinical intervention and public health strategy.

## Conclusion

This study applied the WHO Laboratory Manual (6th Edition, 2021) criteria to characterise semen parameters and their associations in a cohort of men undergoing infertility evaluation in Kumasi, Ghana. The core findings reveal that a significant proportion of men exhibited abnormalities in sperm morphology and, most notably, leukocyte concentration, suggesting that teratozoospermia and leukocytospermia are key features of male infertility in this population. The analysis also identified preliminary, hypothesis-generating associations between certain modifiable lifestyle factors, particularly high sugar consumption, and semen quality. The data suggest a complex aetiology characterised by severe dysfunction within specific subgroups (e.g., high inflammation, poor morphology) rather than a simple, population-wide decline in sperm counts. Our data provides a vital baseline for the region using the latest international standards. It highlights a critical burden of inflammatory or infectious issues, evidenced by the high rate of leukocytospermia, which may be a particular feature of male infertility in this population. These findings underscore the need for targeted clinical investigation into underlying infections and further research into local environmental and lifestyle determinants of male infertility in West Africa.

### Limitations of the study

The study has several limitations that should be considered when interpreting the results.

First, its retrospective cohort design is inherently limited; while it is effective for identifying associations, it cannot establish causality between lifestyle factors and semen parameters. The reliance on pre-existing clinical records also introduced the potential for missing data on certain secondary variables.

Second, the lifestyle data were self-reported by patients during their clinical consultations and are therefore subject to recall bias and social desirability bias, which may affect the accuracy of the reported exposures.

Finally, the study was conducted at a single, private specialist hospital in an urban centre. The patient cohort may therefore have a different socioeconomic, educational, and health-seeking profile compared to the general population, which could limit the generalizability of the findings to rural populations or those who do not access private healthcare.
